# Interspecific diversity in root antioxidative enzyme activities reflect root turnover strategies and preferred habitats in wetland graminoids

**DOI:** 10.1002/ece3.992

**Published:** 2014-02-20

**Authors:** Çağdaş Kera Yücel, Melike Bor, Peter Ryser

**Affiliations:** 1 Department of Biology, Laurentian University935 Ramsey Lake Road, Sudbury, ON, P3E 2C6, Canada; 2 Department of Biology, Science Faculty, Ege UniversityBornova, Izmir, 35100, Turkey

**Keywords:** Plant ecological strategies, plant economics spectrum, plant traits, root senescence

## Abstract

Antioxidant enzymes protect cells against oxidative stress and are associated with stress tolerance and longevity. In animals, variation in their activities has been shown to relate to species ecology, but in plants, comparative studies with wild species are rare. We investigated activities of five antioxidant enzymes – ascorbate peroxidase (APX), catalase (CAT), glutathione reductase (GR), peroxidase (POX), and superoxide dismutase (SOD) – in roots of four perennial graminoid wetland species over a growing season to find out whether differences in root turnover or habitat preferences would be associated with variation in seasonal patterns of antioxidant enzyme activities. The investigated species differ in their root turnover strategies (fine roots senesce in the fall or fine roots survive the winter) and habitat preferences (nutrient-poor vs. productive wetlands). Roots were collected both in the field and from garden-grown plants. Antioxidant enzyme activities were higher and lipid peroxidation rates lower in species with annual root systems, and for species of the nutrient-poor wetland, compared with perennial roots and species of productive wetlands, respectively. There was variation in the activities of individual antioxidant enzymes, but discriminant analyses with all enzymes revealed a clear picture, indicating consistent associations of antioxidant enzyme activities with the type of root turnover strategy and with the preferred habitat. We conclude that antioxidant enzyme activities in plant roots are associated with the species' ecological strategies and can be used as traits for the characterization of the species' position along plant economics spectrum.

## Introduction

The ecological significance of antioxidant enzymes is increasingly being acknowledged in ecological literature (McGraw et al. 2010). These enzymes are important not only for efficient scavenging of reactive oxygen species (ROS) formed as consequence of the diurnal metabolic activities (Van Breusegem et al. 2008), but also in context of stress tolerance of an organism. To date, there are several reports indicating the importance of active antioxidant enzyme defense in plants that is associated with physiological responses and genetic adaptations to stresses such as drought (Türkan et al. 2005), cold (Chen et al. 2006), waterlogging (Sairam et al. 2011), salinity (Bor et al. 2003), light (Streb et al. 1997), high temperatures (Banowetz et al. 2007), P-deficiency (Kandlbinder et al. 2004), toxic metals (Giannakoula et al. 2010), and air pollution (Barnes et al. 1999). Antioxidant enzymes are also associated with the life span of an organism, cumulative oxidative damage being one of the major causes underlying aging process (Harman 1956; Buttemer et al. 2010). In *Drosophila,* longevity has been associated with high levels of antioxidant enzyme activity (Orr and Sohal 1994), and in annual plants, such as *Arabidopsis* or corn, longevity has been shown to be positively associated with resistance to oxidative stress (Kurepa et al. 1998; Procházková et al. 2001; Woo et al. 2004). In plants, aging usually refers to the process of senescence (Thomas 2013), during which the activities of antioxidant enzymes can show different temporal patterns such as an initial increase followed by a decrease, a continuous decrease, or a continuous increase (Procházková and Wilhelmová 2007).

Among animal taxa variations in stress-induced levels of antioxidant enzyme activities are often related to life-history trade-offs and used to explain ecological patterns (Costantini et al. 2010). However, in plants, the majority of research has been carried out with crop plants, annual model species such as *Arabidopsis*, or trees, often addressing genotypic differences in tolerance to environmental stresses. Some work has been performed with extremely stress-tolerant natural species such as plants growing in hot springs (Banowetz et al. 2007) or resurrection plants able to withstand total desiccation (Vicré et al. 2004; Veljovic-Jovanovic et al. 2006), and it has been shown that higher constituent and induced levels of antioxidant enzyme activities are important for plant survival and growth in harsh environments (Bor et al. 2003; Özkur et al. 2009). However, comparative studies in this context with wild plants are rare. Zhou and Zhao (2004) found that among four alpine forage grass species, antioxidant enzyme levels generally increase at the onset of the cold season, but the responses were enzyme-and species-specific.

The purpose of the present work is to investigate to what extent antioxidant enzyme patterns reflect plant functional types differing in seasonal patterns of root mortality and to what extent they reflect the characteristic environment of the species. Graminoid species of temperate wetlands provide a good opportunity for such comparisons, as they combine as a group consistent growth form with wide functional variety (Vernescu and Ryser 2009), occurrence in ecologically contrasting habitats, and strong seasonal patterns in their growth (Bernard and Fitz 1979). Roots of most graminoid species in Northern Ontario wetlands are perennial and show only low mortality during the winter, but roots of some species show annual dieback at the end of the growing season (Ryser and Kamminga 2009). Roots of these species die before the onset of tissue-killing frosts; hence, the dieback has to be regarded as the plant's response to environmental cues, similarly to leaf senescence of deciduous trees. However, it is not clear whether root death can be regarded as programmed senescence. Fisher et al. (2002) did not find any evidence for this in *Phaseolus vulgaris*, which is an annual plant with terminal senescence, whereas Freschet et al. (2010) found evidence of nutrient remobilization in dying fine roots of subarctic perennial species. In northeastern Ontario, species with overwintering roots and species with root dieback in the fall can be found both in productive and in nutrient-poor wetlands.

In order to investigate the association between root antioxidant enzyme activities and root turnover strategies, we compare four species over a growing season: Two species – *Sparganium androcladum* (Engelmann) Morong and *Rhynchospora alba* L. – with roots which senesce in the fall, and two species – *Scirpus microcarpus* J. Presl and C. Presl and *Carex exilis* Dewey – with perennial roots, one species of each root type characteristic of nutrient-poor wetlands and one species characteristic of productive wetlands (Fig. 4). Seasonal measurements were taken on field-grown plants, but as antioxidant enzyme activities are influenced by environmental conditions, we also conducted a common garden experiment to test for existence of inherent interspecific differences in patterns of antioxidant enzyme activity. As there is indication in the literature that longevity is positively associated with antioxidant enzyme activity, we hypothesized that roots which survive the winter have constitutively higher antioxidant enzyme levels, and that toward the end of the season antioxidant enzyme activity would decrease in senescing roots, but increase in the roots which survive the winter.

Among birds, it has been shown that correlations among different antioxidant enzymes show large ecological heterogeneity indicating that conceptualization of single antioxidant enzymes may be too simplistic, and it has been suggested that ecological studies examining antioxidant function should simultaneously use measures of multiple variables (Cohen Z McGraw 2009). Hence, besides investigating the five studied antioxidant enzymes separately, we also analyzed the interspecific differences in the enzyme activities with a discriminant analysis using the combinations of all the five measured antioxidant enzymes.

## Materials and Methods

### Plant material and harvests

Four perennial monocotyledonous graminoid wetland species with contrasting root life spans were used in this study: *Sparganium androcladum* and *Rhynchospora alba* have root systems with a complete mortality in the fall, whereas *Scirpus microcarpus* and *Carex exilis* roots mostly survive the winter (Ryser and Kamminga 2009; Peter Ryser, personal observations). Leaves of all four species senescence for the winter, green leaves being present from mid May to October. *R. alba* overwinters as bulbils, *S. androcladum* as rhizomes. *R. alba* and *C. exilis* were collected along a creek running through a floating fen 50 km northwest of Sudbury (46°41′49.27″N, 81°32′57.20″W; 420 m a.s.l.), *S. androcladum* in a productive marsh along a creek (46°24′22″N, 80°52′36″W; 240 m a.s.l.) and *S. microcarpus* in a productive swamp (46° 21′ 32″N, 80°49′ 44″W; 220 m a.s.l.), 13 and 20 km southeast of Sudbury, respectively. The substrate in which the roots were growing was for *R. alba* sphagnum peat, for *C. exilis* peat and loose organic debris, for *S. androcladum* soft mud and for *S. microcarpus* clay.

**Figure 1:**
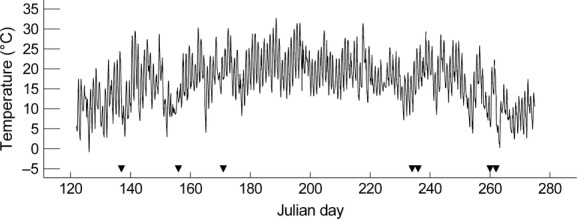
Hourly air temperature at Sudbury Airport from 1 May to 30 September 2012. Harvests are indicated with filled triangles: first field harvest on 17 May and 5 June, second field harvest on 22 and 24 August, third field harvest on 17 and 19 September for the two sites, respectively, and the garden harvest 20 June. All study sites are located within 50 km of the measuring station. Measurement by Environment Canada (http://www.climate.weatheroffice.ec.gc.ca).

Field-grown plants were harvested on three occasions over the season. The first harvest was conducted on 17 May 2012 for *S. androcladum* and *S. microcarpus*, and due to slower spring growth of plants in the fen, on 5 June for *R. alba* and *C. exilis*. The second and third harvests were conducted for *R. alba* and *C. exilis* on 22 August and 17 September, and for *S. androcladum* and *S. microcarpus* on 24 August and 19 September. The study period was frost-free (Fig. 1). Additionally, potted plants grown in an experimental garden were harvested on 20–21 June. For the garden experiment, *R. alba* bulbils were planted on 30 April in a greenhouse in trays filled with peat and transplanted on 14 May in 5-liter (20 cm diameter) pots filled with a mixture of peat and 1.6% composted sheep manure. For the other species, tillers were directly transplanted in the pots on 28 May. The pots were located outdoors in pools filled with 10–20 cm ground water.

*Sparganium androcladum* roots were washed out of the mud in the field and stored on dry ice (first harvest) or ice (second and third harvest) for the transport to the laboratory. Roots of the *S. microcarpus* (clay) and *R. alba* (sphagnum peat of a floating fen) were harvested in the laboratory from two or three monoliths collected in the field and transported to the laboratory on ice. *C. exilis* grew as large tussocks at the edge of a creek running through a floating fen, and the roots were growing in loose organic debris. Parts of the large tussocks were cut with a knife, pulled out of the water and transported to the laboratory on ice for root collection. All collected roots were then frozen in liquid nitrogen and stored in a freezer (−75°C) until the analyses. For collection, roots of the current year's production were used, recognized by the white color of at least of the root tips.

Eight replicate samples were analyzed for each species in each harvest. An exception was SOD, where due to lack of material only 3–5 replicate samples were analyzed per species and harvest.

### Lipid peroxidation

The level of lipid peroxidation in root samples was determined according to Madhava Rao and Sresty (2000) by measuring malondialdehyde (MDA) content which is the end product of lipid peroxidation. Root samples (0.1 g) were homogenized in 0.1% trichloroacetic acid (TCA). The homogenate was centrifuged at 10,000 ***g*** for 5 min at 4°C and 1 mL 0.5% thiobarbituric acid (TBA) in 20% trichloroacetic acid (TCA) was added to 250-*μ*L aliquot of the supernatant. The mixture was incubated at 95°C for 30 min and then cooled in ice bath. The mixture was then centrifuged at 10,000 g for 15 min at 4°C. The MDA content was calculated from the absorbance measured at 532 nm (correction for unspecific turbidity was performed by subtracting the absorbance at 600 nm from this value) using extinction coefficient of 155 mmol^−1^ cm^−1^ and expressed as nmol MDA g^−1^ FW.

### Enzyme analysis

All analyses were performed at 4°C. For protein and enzyme extractions, root samples (0.5 g) were homogenized in 0.05 M sodium phosphate buffer (pH 7.8) containing 1 mM EDTA.Na_2_ and 2% (w/v) polyvinylpolypyrrolidone (PVPP). Homogenates were centrifuged at 14,000 g for 30 min at 4°C, and supernatant was used for protein content and SOD, POX, APX, GR, and CAT enzyme activity assays. Total soluble protein contents were determined according to Bradford (1976) using bovine serum albumin as a standard. All spectrophotometric analyses were conducted on an UV Visible Cary 100 Bio.

Superoxide dismutase (SOD; EC 1.15.1.1) activity was assayed according to the method of Beauchamp and Fridovich (1971), which measures the inhibition in the photochemical reduction in nitroblue tetrazolium (NBT) spectrophotometrically at 560 nm. One unit of enzyme activity was defined as the quantity of SOD required to produce a 50% inhibition of reduction in NBT. The reaction mixture contained 50 mM Na-phosphate buffer (pH 7.8), 33 mM NBT, 10 mM L-methionine, 0.66 mM EDTA, and 0.0033 mM riboflavin. Reactions were carried out at 25°C, under light intensity of about 300 *μ*mol m^−2^ s^−1^ through 10 min.

Peroxidase (POX; EC 1.11.1.7) activity was determined according to the method of Herzog and Fahimi (1973). The reaction mixture contained 3,3‘-diaminobenzidine-tetrahydrochloride dihydrate (DAB) solution containing 0.1% (w/v) gelatine, 150 mM Na-phosphate-citrate buffer (pH 4.4), and 0.6% H_2_O_2_. The increase in the absorbance at 465 nm was monitored for 3 min. One enzyme unit was defined as *μ*mol mL^−1^ H_2_O_2_ decomposed per min.

Ascorbate peroxidase (APX; EC 1.11.1.11) activity was determined according to Nakano and Asada (1981). The assay depends on the decrease in absorbance at 290 nm as ascorbate was oxidized (extinction coefficient of 2.8 mM^−1^ cm^−1^). The reaction mixture contains 50 mM Na-phosphate buffer (pH 7.0), 0.5 mM Ascorbate, 0.1 mM EDTA Na_2_, and 1.2 mM H_2_O_2_. One enzyme unit is defined as mmol mL^−1^ oxidized ascorbate per min.

Catalase (CAT; EC 1.11.1.6) enzyme activity was determined according to Bergmeyer (1970), which measures the decline in the absorbance of H_2_O_2_ at 240 nm. Reaction mixture contained 0.05 M Na-phosphate buffer (pH 7.0) with 1 mM EDTA and 3% H_2_O_2_. Decrease in the absorption was followed for 3 min and *μ*mol H_2_O_2_ destroyed per min was defined as one enzyme unit.

Glutathione reductase (GR; EC 1.6.4.2) activity was quantified according to the method of Foyer and Halliwell (1976), which depends on the rate of decrease in the absorbance of oxidized glutathione (GSSG) at 340 nm. Reaction mixture contained 25 mM Na-phosphate buffer (pH 7.8), 5 mM GSSG, 1.2 mM NADPH.Na_4_. Activity of GR was calculated from reduction in GSSG level using extinction coefficient 6.2 mM^−1^ cm^−1^. One enzyme unit was defined as *μ*mol mL^−1^ oxidized GSSG per min.

### Root vitality

Root vitality was determined after vital staining with triphenyltetrazolium chloride (TTC; Larcher 1969). Roots were cut into 10-to 15-mm pieces and incubated at 30°C in 0.3 (w/v) TTC and 10 mM glucose solution for 36–48 h. Water-insoluble red formazan, formed from the reduction in TTC by dehydrogenase enzymes (Ruf and Brunner 2003) turns living roots red or pink, the percentage of which was determined from 100 roots per plant crossing grid lines when spread on a petri dish.

### Statistical analyses

Statistical analyses were conducted with SyStat 5.2.2 for MacIntosh and SyStat 12. To attain normality, antioxidant enzyme and MDA data were log-transformed. Levels of each antioxidant enzyme and MDA were analyzed separately using ANOVAs with species as an independent factor, and in case of the field-collected plants, the time of collection as another independent factor. Significance levels between species of different root turnover type within each wetland type, and between species with the same root turnover type, but grown on different wetland type were tested using Bonferroni-corrected contrasts.

Differences among the species with respect of all five measured antioxidant enzymes were tested with linear discriminant analyses. To test species differences, we used the original dataset, but to attain more clarity in the graphical presentation of the field-collected data, we conducted another analysis with species averages at each harvest.

## Results

Staining with TTC showed that roots were alive to more than 90% in all harvests but the last one. In the September harvest, 69 ± 1%, 80 ± 3%, 83 ± 1% and 75 ± 2% of the roots of *C. exilis*,*R. alba*,*S. microcarpus* and *S. androcladum* were alive, respectively (mean ± SE, *n* = 5). One sample of *S. androcladum* with only 7% vitality was not included in the calculation of the averages.

**Figure 2:**
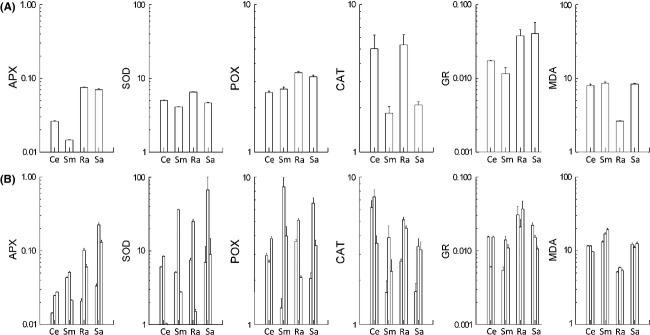
Levels of antioxidant enzymes APX, SOD, POX, CAT, and GR (unit mg protein^−1^) and lipid peroxidation (MDA; nmol g fresh mass^−1^) levels in roots of (A) garden-grown (harvested in June 2012) and (B) field-collected plants (the three bars for each species and antioxidant enzyme refer to the three harvests in May/June, August, and September; for dates see Fig. 1.)

Activities of all five measured antioxidant enzymes in roots showed highly significant interspecific variation both for the plants grown in common garden and harvested in June and for the field-grown plants harvested three times in course of the season (Table 1; Fig. 2). Among the species of each wetland type, those with annual roots generally had higher levels of antioxidant enzymes than those with perennial roots (Table 2). Results were similar for garden-grown and field-collected plants, the differences in both being significant for APX and GR. Also the activities of SOD and POX were higher in the annual roots, but the difference in SOD was significant for garden-grown plants only and the difference in POX was significant for field-collected plants only. As an exception to the generally higher levels of antioxidant enzymes in plants with annual roots, CAT levels in field-collected roots were significantly lower in *R. alba* compared with *C. exilis* (Table 2).

**Table 1:** ANOVAs of antioxidant enzyme levels in roots of garden-grown and field-collected plants of the four species *C. exilis*,*R. alba, S. microcarpus,* and *S. androcladum*. Plants in the field were collected in May/June, August and September. Species (Sp) was an independent factor for all analyses. For field-collected plants, the time of harvest (H) was an additional independent factor.

	*N*	*R*^*2*^	*F* (Sp)	*F* (H)	*F* (H × S)
Garden					
APX	27	0.888	61.0***	–	–
SOD	15	0.763	11.8***	–	–
POX	26	0.681	15.6***	–	–
CAT	27	0.654	14.5***	–	–
GR	27	0.539	9.0***	–	–
MDA	26	0.823	34.2***	–	–
Field					
APX	96	0.763	39.6***	43.9***	10.7***
SOD	41	0.963	72.2***	198***	21.1***
POX	95	0.907	3.4*	186***	72.1***
CAT	95	0.672	30.5***	22.1***	5.3***
GR	95	0.545	18.8***	0.9	6.9***
MDA	92	0.776	84.9***	1.1	3.0*

*F*-values and significance levels given:

*** *P* < 0.001,

**P < 0.01,

* *P* < 0.05.

**Table 2:** Comparisons between species of contrasting root turnover types within each wetland type, comparisons between species with similar root turnover types, but growing in different types of a wetlands, and comparisons between the harvests. Comparisons are expressed as ratios of the least squares means of antioxidant enzyme levels between species with annual and perennial roots (*R. alba*/*C. exilis*;*S. androcladum*/*S. microcarpus*), between species of the nutrient-poor wetland and nutrient-rich wetlands (*C. exilis*/*S. microcarpus*;*R. alba*/*S. androcladum*), and between the different harvests of field-grown plants (August/June; September/August). The least squares means are based on ANOVAs presented in Table 1 with significance of the differences between the numerator and denominator in the ratios being calculated using Bonferroni-corrected contrasts.

	Ra/Ce	Sa/Sm	Ce/Sm	Ra/Sa	Aug/Jun	Sep/Aug
Garden						
APX	2.9***	4.8***	1.9*	1.1	–	–
SOD	1.3+	1.1	1.2	1.4**		
POX	1.4***	1.2***	0.95	1.1	–	–
CAT	1.0	1.3	3.1**	2.5***	–	–
GR	2.1	4.5**	2.2	1.0	–	–
MDA	0.33***	0.99	0.94	0.32***	–	–
Field						
APX	2.3***	3.0***	0.67*	0.51***	3.2***	0.59***
SOD	1.7***	2.0***	0.53***	0.46***	4.3***	0.85
POX	1.1	1.0	0.9+	0.9	2.3***	0.61***
CAT	0.7***	1.2	2.4***	1.5**	1.8***	0.66***
GR	2.6***	1.5+	1.2	2.0***	0.85	1.2
MDA	0.50***	0.71***	0.67***	0.47***	1.1	1.0

Significance levels and trends are given:

*** *P* < 0.001,

** *P* < 0.01,

* *P* < 0.05,

+*P* < 0.10.

When comparing species with similar types of root turnover patterns across the wetland types, CAT levels were clearly higher for species of nutrient-poor wetlands compared with species of nutrient-rich wetlands both in garden-grown and field-collected roots (Table 2). In garden-grown plants, there was some indication this being the case for APX and SOD as well, but in the field-harvested roots, APX and SOD levels were 30–50% lower in species of the floating fen.

Lipid peroxidation in field-grown plants was higher in roots of species with perennial roots and higher in species of productive wetlands compared with species of nutrient-poor wetlands. Despite their statistical significance, the differences were small except for the distinctly lower values in *R. alba* (Fig. 2; Table 2). In garden-grown plants, the only significant difference was the lower value in *R. alba* roots compared with other species.

Levels of all enzymes but GR varied significantly across the seasons (Table 1). The highest enzyme activities were generally found in August. Species × Harvest interaction was significant for all enzymes, the most obvious interspecific difference in seasonal pattern being shown by the GR levels which for *C. exilis* and *R. alba* were the lowest in August. Lipid peroxidation did not show any significant seasonal variation, but there was a weak Species × Harvest interaction due to a slight increase in MDA content in *S. microcarpus* during the season.

**Figure 3:**
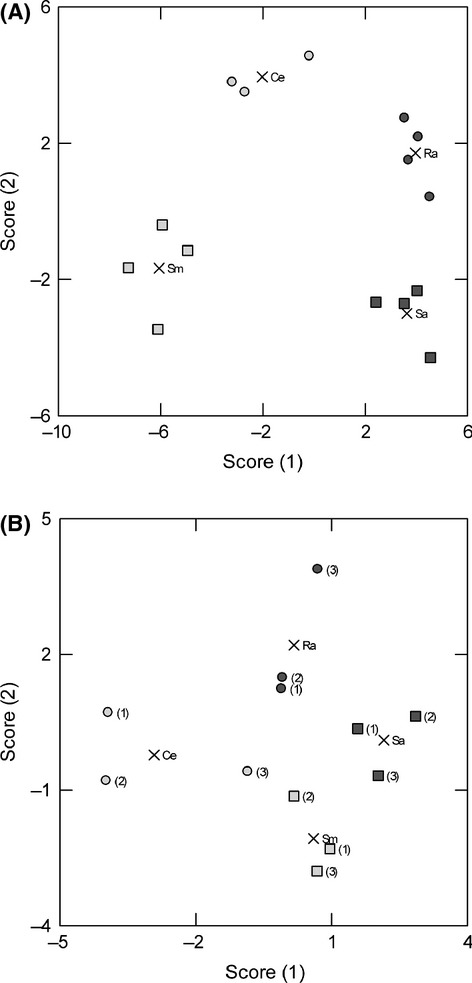
Canonical scores of the investigated species along the two first discriminant functions in discriminant analyses of (A) garden-grown plants and of (B) three harvests of field-collected plants. For the analysis of field-collected data, species averages in each harvest were used. The canonical discriminant functions for garden-grown plants are F1 = 1.09 APX+0.66 POX+0.61 CAT-0.10 GR-0.65 SOD and F2 = 0.12 APX-1.58 POX+0.92 CAT-0.06 GR+1.26 SOD. For the field-grown plants, the functions are F1 = 0.93 APX-0.20 POX-1.25 CAT+0.32 GR+0.59 SOD and F2 = 0.75 APX-0.79 POX+0.63 CAT +1.21 GR-0.33 SOD. Species with annual roots have filled symbols (Ra = *Rhynchospora alba*, Sa = *Sparganium androcladum*), species with perennial roots open symbols (Ce = *Carex exilis*, Sm = *Scirpus microcarpus*). Circles: species of nutrient-poor wetlands (Ce, Ra); squares: species of nutrient-rich wetlands (Sm, Sa). The harvest is indicated in parentheses (1 = June; 2 = August; 3 = September). Species centroids are indicated with a cross.

Discriminant analyses based on the levels of all five measured antioxidant enzymes in roots clearly differentiated among the four species, both the garden-grown and the field-grown plants (Wilks' Lambda, *P* < 0.001; Fig. 3). For garden-grown plants, all specimens (100%) were correctly identified to species based on their antioxidant enzyme data, and for field-grown plants, this was the case for all specimens but two (one *S. microcarpus* and one *S. androcladum* were misidentified). Enzymes that contributed most to the discrimination were for the field-collected plants CAT, GR, and APX, and for the garden-grown plants APX, POX, and CAT (Table 3). Interspecific differences with respect to the two first discriminant functions were consistently related to the type of root turnover pattern and to the leftacteristic habitat of the species (Fig. 3). In garden-grown plants, discriminant function 1 differentiated between species with annual roots and those with perennial roots, whereas discriminant function 2 differentiated between the species of the different kinds of wetlands. For field-grown plants, the two first discriminant functions contributed to about equal amounts both to differentiation between species with different root types and different habitat preferences.

**Table 3:** Results of the Discriminant Analyses based on five antioxidant enzyme levels in roots of the four investigated species.

	Garden-grown plants (*N* = 15)		Field-collected plants (*N* = 41)	
	Wilks' lambda = 0.003, df = (5, 3, 11), *F* = 9.9***		Wilks' lambda = 0.17, df = (5, 3, 37), *F* = 5.5***	
	*F*-to remove	Tolerance	*F*-to remove	Tolerance
APX	7.09	0.65	3.35	0.69
POX	5.65	0.26	1.26	0.43
CAT	4.19	0.57	13.91	0.63
GR	0.07	0.83	5.37	0.83
SOD	2.42	0.27	1.81	0.38

*** *P* < 0.001.

## Discussion

The data demonstrate that antioxidant enzyme patterns are species-specific and associated with the ecological strategy of the species. They also support findings of Cohen and McGraw (2009) that interspecific relationships can be best studied using multiple measures. In our study, most antioxidant enzymes showed large seasonal variation, but when regarding all the five antioxidant enzymes over the entire growing season, the patterns were clearly distinct for the four studied species. Moreover, the observed antioxidant enzyme profiles were consistently associated with functional and ecological leftacteristics of the species: within each habitat type, the difference between the two root turnover types – species with annual roots vs. species with perennial roots – was consistent, and within each turnover type, the difference between the ecological preferences – productive wetlands vs. low-productive wetlands – was comparable. The small number of species in this study limits the extent of generalizations and functional interpretations that can be made based on this data. Nevertheless, the distinct differences in patterns of antioxidant enzyme activities among these four species, and the consistent association of these differences both in the field-and garden-collected data with species ecology strongly indicate that consistent ecological patterns of antioxidant types exist among plant species. Such relationships have previously been found for bird species (Cohen and McGraw 2009).

In contrast to our hypothesis, however, annual roots had generally higher antioxidant enzyme activities compared with perennial roots. This contrasts data for *Arabidopsis* and *Zea mays,* in which long-lived genotypes have been shown to have higher levels of antioxidant enzymes (Procházková et al. 2001; Woo et al. 2004). The different results in our study may be related to the monocarpic nature of *Arabidopsis* and *Zea mays*, the above-mentioned investigations referring to genetic variation in the time of the terminal senescence of the whole plant, whereas our data refer to presence or absence of seasonal organ senescence in perennial plants. The generally higher antioxidant enzyme activity of the species with short-lived roots could be understood in context of their potentially higher metabolic rates. Reactive oxygen species are by-products and regulators of many metabolic processes, such as in mitochondrial respiration and photosynthesis (Apel and Hirt 2004), and species with higher metabolic rates may require higher constitutive and induced levels of antioxidant enzymes as protection, as found, for example, in lichens (Beckett et al. 2003). We measured antioxidant enzyme activity per fresh mass, and the annual roots do have a lower dry-mass-to-fresh-mass ratio than the winter-surviving roots (Dominique Gagnon, unpublished data), indicating a higher proportion of a cytosolic component in tissue, and hence, a higher metabolic activity per fresh mass (Roderick et al. 1999). The higher activity of several antioxidant enzymes and lower degree of lipid peroxidation in the species of the nutrient-poor fen, compared with those of the more productive wetlands, is in agreement with such findings for plants adapted to harsh environmental conditions such as *Beta maritima* (Bor et al. 2003) and *Capparis ovata* (Özkur et al. 2009).

Our second hypothesis, that annual and perennial roots would differ in their seasonal variation in antioxidant enzyme activities especially in the fall when annual roots are heading toward senescence and perennial roots are preparing for the winter, was not clearly confirmed. Most species showed decreasing antioxidant enzyme activity in the fall. Nevertheless, *C. exilis*, probably the most stress-tolerant of our species judging by its scleromorphic leaves showed the least seasonal variation in its enzyme activities and even a late-season increase in some of the enzymes. One also has to keep in mind that senescence is a complex process and for leaf senescence in *A. thaliana,* it was been reported that ROS-related responses may greatly vary within a time scale of days (Breeze et al. 2011).

The absence of seasonal variation in lipid peroxidation, and the remarkably low interspecific differences, except for *R. alba*, supports the notion of a protective significance of the antioxidant enzymes and that the seasonal and interspecific variation in the enzyme levels were functional responses to variation in levels of oxidative stress.

We conclude that antioxidant enzyme patterns are useful traits for ecological leftacterization of plant species. Even though the interpretation of the levels of individual antioxidant enzymes was not always straightforward, the analysis including all five studied enzymes revealed clear and consistent distinctions between species with different root turnover strategies and different habitat preferences, across harvests and both in the field and in a common garden experiment. This supports the conclusion of Cohen and McGraw (2009) of the importance to include a multitude of variables in the investigations when considering the ecological significance of antioxidant enzymes. The association of root antioxidant enzyme activities with root life span and habitat productivity indicates that in a similar manner than known for leaf traits (Shipley et al. 2006), traits related to root physiology can be used to leftacterize plants along an economics spectrum.

## Figures and Tables

**Figure A1:**
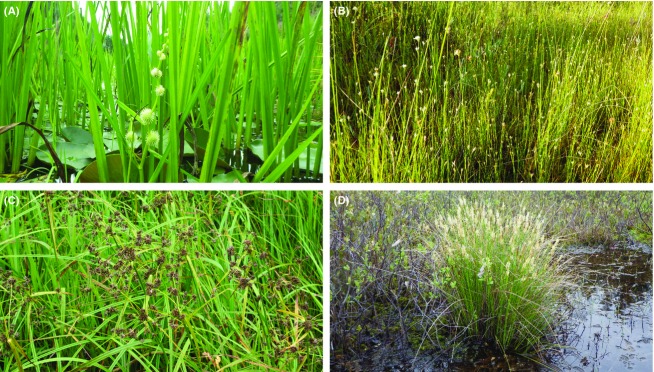
Species studied: (A) *Sparganium androcladum,* (B) *Rhynchospora alba,* (C) *Scirpus microcarpus, and* (D) *Carex exilis*.

## References

[b1] Apel K, Hirt H (2004). Reactive oxygen species: metabolism, oxidative stress, and signal transduction. Annu. Rev. Plant Biol.

[b2] Banowetz GM, Azevedo MD, El-Nashaar HM, Martin RC, Stout RG (2007). Temperature-induced increase in cellular chelating potential associated with reduced thermotolerance. J. Therm. Biol.

[b3] Barnes J, Bender J, Lyons T, Borland A (1999). Natural and man-made selection for air pollution resistance. J. Exp. Bot.

[b4] Beauchamp C, Fridovich I (1971). Superoxide dismutase: improved assays and an assay applicable to acrylamide gels. Anal. Biochem.

[b5] Beckett RP, Minibayeva FV, Vylegzhanina NN, Tolpysheva TT (2003). High rates of extracellular superoxide production by lichens in the suborder Peltigerineae correlate with indices of high metabolic activity. Plant, Cell Environ.

[b6] Bergmeyer N (1970). Methoden der enzymatischen analyse.

[b7] Bernard JM, Fitz ML (1979). Seasonal changes in above-ground primary production and nutrient contents in a central New York *Typha glauca* ecosystem. Bull. Torrey Bot. Club.

[b8] Bor M, Özdemir F, Türkan I (2003). The effect of salt stress on lipid peroxidation and antioxidants in leaves of sugar beet *Beta vulgaris* L. and wild beet *Beta maritima* L. Plant Sci.

[b9] Bradford MM (1976). A rapid and sensitive method for the quantitation of microgram quantities of protein utilizing the principle of protein-dye binding. Anal. Biochem.

[b10] Breeze E, Harrison E, McHattie S, Hughes L, Hickman R, Hill C (2011). High-resolution temporal profiling of transcripts during *Arabidopsis* leaf senescence reveals a distinct chronology of processes and regulation. Plant Cell.

[b11] Buttemer WA, Abele D, Costantini D (2010). From bivalves to birds: oxidative stress and longevity. Funct. Ecol.

[b12] Chen Y, Zhang M, Chen T, Zhang Y, An L (2006). The relationship between seasonal changes in anti-oxidative system and freezing tolerance in the leaves of evergreen woody plants of *Sabina*. S. Afr. J. Bot.

[b13] Cohen AA, McGraw KJ (2009). No simple measures for antioxidant status in birds: complexity in inter-and intraspecific correlations among circulating antioxidant types. Funct. Ecol.

[b14] Costantini D, Rowe M, Butler MW, McGraw KJ (2010). From molecules to living systems: historical and contemporary issues in oxidative stress and antioxidant ecology. Funct. Ecol.

[b15] Fisher MCT, Eissenstat DM, Lynch JP (2002). Lack of evidence for programmed root senescence in common bean (*Phaseolus vulgaris*) grown at different levels of phosphorus supply. New Phytol.

[b16] Foyer CH, Halliwell B (1976). The presence of glutathione and glutathione reductase in chloroplasts: a proposed role in ascorbic acid metabolism. Planta.

[b17] Freschet GT, Cornelissen JHC, Aerts RSP, van Logtestijn R (2010). Substantial nutrient resorption from leaves, stems and roots in a subarctic flora: what is the link with other resource economics traits?. New Phytol.

[b18] Giannakoula A, Moustakas M, Syros T, Yupsanis T (2010). Aluminum stress induces up-regulation of an efficient antioxidant system in the Al-tolerant maize line but not in the Al-sensitive line. Environ. Exp. Bot.

[b19] Harman D (1956). Aging: a theory based on free radical and radiation chemistry. J. Gerontol.

[b20] Herzog V, Fahimi H (1973). Determination of the activity of peroxidase. Anal. Biochem.

[b21] Kandlbinder A, Finkemeier I, Wormuth D, Hanitzsch M, Dietz KJ (2004). The antioxidant status of photosynthesizing leaves under nutrient deficiency: redox regulation, gene expression and antioxidant activity in *Arabidopsis thaliana*. Physiol. Plant.

[b22] Kurepa J, Smalle J, Inzé M, Van Montagu D (1998). Oxidative stress tolerance and longevity in *Arabidopsis*: the late-flowering mutant gigantea is tolerant to paraquat. Plant J.

[b23] Larcher W (1969). Anwendung und Zuverlässigkeit der Tetrazoliummethode zur Feststellung von Schäden in pflanzlichen Geweben. Mikroskopie.

[b24] Madhava Rao KV, Sresty TVS (2000). Antioxidative parameters in the seedlings of pigeonpea (*Cajanus cajan* L. Millspaugh) in response to Zn and Ni stresses. Plant Sci.

[b25] McGraw KJ, Cohen AA, Costantini D, Horak P (2010). The ecological significance of antioxidants and oxidative stress: a marriage between mechanistic and functional perspectives. Funct. Ecol.

[b26] Nakano Y, Asada K (1981). Hydrogen peroxide is scavenged by ascorbate-specific peroxidase in spinach chloroplasts. Plant Cell Physiol.

[b27] Orr WC, Sohal RS (1994). Extension of life-span by overexpression of superoxide dismutase and catalase in *Drosophila melanogaster*. Science.

[b28] Özkur O, Özdemir F, Bor M, Turkan I (2009). Physiological and antioxidant responses of the perennial xerophyte *Capparis ovata* Desf. to drought. Environ. Exp. Bot.

[b29] Procházková D, Wilhelmová N (2007). Leaf senescence and activities of the antioxidant enzymes. Biol. Plant.

[b30] Procházková D, Sairam RK, Srivastava GC, Singh DV (2001). Oxidative stress and antioxidant activity as the basis of senescence in maize leaves. Plant Sci.

[b31] Roderick ML, Berry S, Noble IR, Farquhar GD (1999). A theoretical approach to linking the composition and morphology with the function of leaves. Funct. Ecol.

[b32] Ruf M, Brunner I (2003). Vitality of tree fine roots: reevaluation of the tetrazolium test. Tree Physiol.

[b33] Ryser P, Kamminga AT (2009). Root survival of six cool-temperature wetland graminoids in autumn and early winter. Plant Ecol. Divers.

[b34] Sairam RK, Dharmar K, Lekshmy S, Chinnusamy V (2011). Expression of antioxidant defense genes in mung bean (*Vigna radiata L*.) roots under water-logging is associated with hypoxia tolerance. Acta Physiol. Plant.

[b35] Shipley B, Lechowicz MJ, Wright I, Reich PB (2006). Fundamental trade-offs generating the worldwide leaf economics spectrum. Ecology.

[b36] Streb P, Tel-Or E, Feierabend J (1997). Light stress effects and antioxidative protection in two desert plants. Funct. Ecol.

[b37] Thomas H (2013). Senescence, ageing and death of the whole plant. New Phytol.

[b38] Türkan I, Bor M, Özdemir F, Koca H (2005). Differential responses of lipid peroxidation and antioxidants in the leaves of drought-tolerant *P. acutifolius* Gray and drought-sensitive *P. vulgaris* L. subjected to polyethylene glycol mediated water stress. Plant Sci.

[b39] Van Breusegem F, Bailey-Serres J, Mittler R (2008). Unraveling the tapestry of networks involving Reactive Oxygen Species in plants. Plant Physiol.

[b40] Veljovic-Jovanovic S, Kukavica B, Stevanovic B, Navari-Izzo F (2006). Senescence-and drought-related changes in peroxidase and superoxide dismutase isoforms in leaves of *Ramonda serbica*. J. Exp. Bot.

[b41] Vernescu C, Ryser P (2009). Constraints on leaf structural traits in wetland plants. Am. J. Bot.

[b42] Vicré M, Farrant JM, Driouich A (2004). Insights into the cellular mechanisms of desiccation tolerance among angiosperm resurrection plant species. Plant, Cell Environ.

[b43] Woo HR, Kim JH, Nam HG, Lim PO (2004). The delayed leaf senescence mutants of *Arabidopsis**ore1**ore3*, and *ore9* are tolerant to oxidative stress. Plant Cell Physiol.

[b44] Zhou R, Zhao H (2004). Seasonal pattern of antioxidant enzyme system in the roots of perennial forage grasses grown in alpine habitat, related to freezing tolerance. Physiol. Plant.

